# Factors Associated with Higher Body Mass Index, Weight Concern, and Weight Gain in a Multinational Cohort Study of Smokers Intending to Quit

**DOI:** 10.3390/ijerph6030943

**Published:** 2009-03-02

**Authors:** Henri-Jean Aubin, Ivan Berlin, Elisheva Smadja, Robert West

**Affiliations:** 1 Assistance Publique – Hôpitaux de Paris, Hôpital Paul Brousse, 94804 Villejuif Cedex, France; Centre Hospitalier Emile Roux, 94456 Limeil-Brévannes Cedex, France; INSERM U669; 2 Centre Hospitalier Universitaire Pitié Salpétrière, Assistance Publique – Hôpitaux de Paris, 47–83 boulevard de l'Hôpital 75651 Paris Cedex, France; E-Mail: ivan.berlin@psl.aphp.fr; 3 Sanofi-Aventis Recherche & Développement, 182, av. de France, 75013 Paris, France; E-Mail: Elisheva.Smadja@sanofi-synthelabo.com; 4 Cancer Research UK Health Behaviour Unit, Department of Epidemiology and Public Health, University College London, Brook House, 2–16 Torrington Place, London WC1E 6BT, UK; E-Mails: robert.west@ucl.ac.uk

**Keywords:** Smoking cessation, body mass index, weight concern

## Abstract

The ATTEMPT cohort study is multi-national, longitudinal study of smokers intending to quit recruited in the U.S.A., Canada, U.K. and France. Data on demographics, medical history, body mass index (BMI), weight concerns and smoking status were collected at baseline and after six months. A total of 2,009 subjects provided data at baseline and 1,303 at six months. High baseline BMI was associated with recent quit attempts, high weight concerns and high cigarette consumption. Weight gain was associated with low income, being single and number of cigarette-free days, but not with baseline weight concerns and confidence in preventing weight gain. Quit attempts were more frequent in subjects with a high baseline BMI and low weight concerns.

## 1. Introduction

Although the health benefits of giving up smoking are unequivocal [1,2] and encouraging smoking cessation is an important public health goal, willingness to quit and successful cessation are often compromised by beliefs about relative benefit and risks that may or may not be founded. One of these beliefs, especially in women, relates to fear of weight gain after stopping smoking [3,4]. To a certain extent, these fears are supported by documented associations between weight concerns, weight gain, and smoking cessation. Understanding and challenging these beliefs has become increasingly important with the emerging epidemic of obesity [5,6] growing public awareness and stigmatisation of weight gain.

Several large epidemiological studies have provided evidence for reduced body weight in smokers. For example, the National Health and Nutrition Examination Survey II, performed in the U.S.A. between 1976 and 1980, found that smokers weighed on average three kilograms less than non-smokers, and were leaner, as determined by body mass index (BMI); these differences were more pronounced in women than in men [7,8]. Similar results have been reported in epidemiological surveys performed in other countries [9–11].

In addition, there is relatively consistent evidence that quitting smoking is frequently followed by an increase in weight [4,12–15]. Such weight gain is thought to occur in around 80% of individuals who give up smoking [2] and is most marked in the first six months after quitting, and then tends to stabilise [13]. The extent of weight gain is variable, but has generally been found to be around six kilograms over a year or less [13,14,16–18]. It is noteworthy that smoking and obesity have been shown to have additive and independent deleterious effects on mortality [19,20].

A number of factors have been postulated to be associated with an increased relative risk of gaining weight upon smoking cessation, including female gender [18], race (higher risk in Blacks [18,21]), low BMI [16,22], past maximum body weight, high previous cigarette consumption [13,18,22,23] high salivary cotinine concentrations [16], concerns about gaining weight [24], low alcohol consumption [16], lack of physical activity [22] and dieting [13,25].

Concerns about weight gain may be an important deterrent to stopping smoking [3,26–30]. The probability of attempting to stop smoking appears to be lower in subjects reporting smoking-related weight concerns [3,26,28], although general weight concerns may be associated with a higher probability of quitting, perhaps due to higher health awareness [3,31–33]. The development of a rating scale to assess specific smoking-related weight concerns [24] has provided a useful instrument to distinguish between specific and general weight concerns. However, it should be noted that other confounding variables, particularly nicotine dependence, may influence the relationship between weight concerns and cessation attempts [3,31]. In addition, subjects reporting high smoking-related weight concerns report more severe tobacco withdrawal symptoms [34].

Smoking-related weight concerns are significantly more frequent in women than in men [17,28,35,36]. Factors related to high weight concerns with respect to smoking include frequent dieting [28], high nicotine dependence [3,29] and high cigarette consumption [29]. Conflicting results have been obtained concerning the association between actual weight and smoking-related weight concerns [28,29].

Although it might be expected that weight gain may be associated with less successful outcome of smoking cessation attempts, due to relapse to smoking in an attempt to regain weight control, paradoxically this does not appear to be the case. Most studies [13,23,33,37–40], but not all [24], have shown that long-term abstinence rates are no lower, if not higher, in subjects who gain weight.

However, many of the studies investigating the relationship between smoking-related weight concerns and smoking cessation attempts have been hampered by low sample-size, retrospective data collection or the absence of a specific instrument to measure these concerns. For this reason, we have wanted to re-assess this issue in a large multinational sample of subjects wishing to stop smoking in whom data on weight concerns could be collected prospectively using the Borrelli scale for smoking-related weight concerns [24]. The ATTEMPT cohort study provides such an opportunity. The ATTEMPT cohort is an observational, multi-national, longitudinal study of smokers intending to quit identified from Internet panels in four countries, who were followed quarterly. The principal objectives of this study were to describe the natural history and patterns of smoking cessation, to explore the influence of weight change and craving on the smoking cessation process and to describe the effects of smoking cessation on short-term health outcomes and medical resource utilisation. A description of the aims, scope and methods of the ATTEMPT study have recently been published elsewhere [41]. The objectives of the present analysis were to identify factors associated with high BMI, weight concern, and confidence in preventing weight gain among smokers at baseline, to identify predictors of significant weight gain (≥5%) over a six-month study period and to describe the influence of BMI and weight concerns on the smoking cessation attempts.

## 2. Methods

### 2.1. Study Design

ATTEMPT is a prospective longitudinal cohort study of cigarette smokers who intend to quit smoking within the next three months performed in the U.S.A., Canada, U.K., and France. Data are. collected quarterly using an internet-based questionnaire. The ATTEMPT study was initiated in the spring of 2003. The aims and methodology of the ATTEMPT study have been described in detail elsewhere [41].

### 2.2. Entry Criteria

The study included regular smokers, smoking on average at least five cigarettes per day, aged between 35 and 65 years, who planned to quit smoking during the following three months. Subjects were required to have regular access to an Internet service. Only one participant in the study was allowed per household. Because of practical limitations of standardized commercially available scales, participants weighing 300 pounds (or 135 kg) or more at baseline were excluded.

### 2.3. Recruitment

Study participants were identified from existing Internet panels in the U.S., Canada, the U.K. and France. A random sample of panel members who met the age, country, and participation criteria were invited to participate via e-mail. In the U.S., invitations were sent to a panel of pre-identified smokers. In all other countries, smoker panels were not available, so invitations were sent to members of the general population panels. The e-mail directed panelists to the online screener questionnaire to determine study eligibility.

### 2.4. Data Collection

Data were collected using a questionnaire administered over the Internet at baseline and quarterly thereafter. Data used in the present analysis come from the baseline and six-month follow-up interviews. The average time required for participants to complete the survey was 25 minutes. The questionnaire was originally developed and validated in American English and subsequently translated into British English and French.

Data were collected on demographics, body mass index (BMI), smoking status, history of smoking and quit attempts, weight concerns and confidence regarding weight changes related to smoking cessation, nicotine dependence and quality of life. In addition, further data on medical history, withdrawal symptoms, craving, healthcare resource utilisation, physical exercise, smoking-related treatments and economic variables were also collected, but these were not used in the present analysis.

Weight was determined by the participants themselves using a standardised body weight scale sent to all participants at the time of inclusion. BMI in kg/m^2^ was calculated retrospectively from self-reported weight and height data. Nicotine dependence was estimated using the Fagerström Test of Nicotine Dependence (FTND) score [42], post-cessation weight concerns and confidence in preventing weight gain using the Borrelli scale [24], ranging from 1 to 10, with a higher score indicating more concernor more confidence, and quality of life using the EQ-5D scale [43].

In order to validate the self-reported information about the weight, a random sample of subjects was selected in the US for in-home assessment of weight after the baseline, the 3-month and the 6-month follow-up &assessment. The participant’s consent was obtained at the time of the baseline assessment but it did not constitute an exclusion criteria. The correlation between the self-reported weight and the weight reported online was then evaluated [41].

### 2.5. Ethics

Panellists who qualified for the ATTEMPT Study were required to provide on-line informed consent to participate in the study. The study was approved by RTI’s Institutional Review Board (IRB). The organisation conducting the Internet surveys abides by the principles set forth in the Safe Harbor framework, as set forth by the U.S. Department of Commerce, regarding the collection, use, and retention of data from the European Union and is a licensee of the TRUSTe Privacy Program.

### 2.6. Statistical Analysis

Subjects whose reported weight varied by more than thirty kilograms between two evaluations were excluded from the analysis. Such a weight change was considered to be unlikely and to possibly reflect an error in reporting weight value. For continuous variables, group differences were assessed using analysis of variance (*ANOVA*) whereas, for categorical variables, the χ^2^ test was used. BMI, weight concerns and confidence were either analysed as continuous or categorical variables. When categorised into two groups, median values were used as cut-offs. For the marital status variable, married and living with a partner were categorised together, and opposed to being single. Income and education were categorised into three groups. Income cut-offs were chosen in each country in order to built 3 groups roughly equivalent in size. Factors associated with high BMI, weight concerns and confidence scores were assessed using stepwise logistic regression. In a first step, univariate analyses on baseline demographics, weight and smoking variables have been displayed. Those variables significantly associated with the independent variable at a probability threshold of 10% were then selected for the multivariate model and odds ratios with 95% confidence limits were calculated. Statistical analyses were generated using SAS for Windows software (Version 8.02, SAS Institute, Cary, NC).

## 3. Results

### 3.1. Study Participants

An e-mail invitation to participate in the ATTEMPT study was sent to 95,183 Internet panel members of whom 13,833 (15%) completed the screener survey. Participants were enrolled on a first-come first-served basis until the target sample size in each country was reached. A total of 2,009 subjects were enrolled, 1,400 in the U.S.A., 208 in Canada, 201 in France and 200 in the U.K. Twenty-three subjects were excluded because they reported a weight change of > 30 kg during the six month follow-up period, leaving a final sample size of 1,986 subjects (1,383 in the U.S.A., 206 in Canada, 197 in France and 200 in the U.K.). Of this initial sample, six-month follow-up data was provided by 1,284 subjects (64.8%) [931 in the U.S.A. (67.3%), 150 in Canada (72.8%), 93 in France (47.2%) and 110 in the U.K. (55.0%)]. Lost to follow-up subjects did not differ significantly from responders at six months for gender, country and age. They did however differ for FTND, lower in lost to follow-up subjects (p<0.01) and for BMI, higher in lost to follow-up subjects (p<0.01).

### 3.2. Demographics

The demographic characteristics of the study cohort at baseline are displayed in Table 1. In summary, the mean age of the study cohort was 48 years, with a similar overall representation of men and women. However, in France, males were somewhat over-represented (63%). European participants were somewhat younger overall than their North American counterparts. The majority of participants were white (90%), especially in France and U.K., and a high proportion were single (37%, including never married, divorced, separated, and widowed) and educated beyond the secondary level (72%). Half of the subjects were employed full time.

### 3.3. Smoking History and Outcome

The proportion of heavy smokers (≥ 20 cigarettes a day) was 45% in men and 40% in women, and higher in the participants in the U.S.A. (p<0.001). As seen in Table 2, the average nicotine dependence score on the Fagerström test was 5.2 (± 2.3), with this parameter again being higher in subjects in the USA (p<0.0001). At baseline, 95% of the sample had made a previous attempt to stop smoking. However, only one third had made an attempt in the three months preceding inclusion. During the six-month follow-up period, 715 (55.7%) of the 1284 subjects had made at least one attempt to give up. Data for a quit attempt during the follow-up was missing for 22 subjects (1.7%).

### 3.4. Weight

Across all countries, the mean weight at baseline was 89 (± 19) kg in men and 75 (± 19) kg in women, with participants from France weighing around 10 kg less than those from other countries. This corresponded to a BMI of 28 (± 5.8) for both men and women. Mean scores on the Borrelli scale for weight concerns were 5.0 (± 2.1) in men and 6.1 (± 2.3) in women, and were much higher in France. Mean scores indicating participants’ confidence to prevent weight gain during smoking cessation evaluated on a Likert-scale (range from 1 to 10) were 6.0 (± 2.1) in men and 5.2 (± 2.2) in women, and lower in participants from the U.K. During the six-month follow-up period, 14% of men and 19% of women gained 5% or more of their body weight, with a lower rate in U.S.A.

Self-reported weight and observed weight (checked by in-home visits) were found to comparable at month 3 (mean difference= −0.2 ±1.8 kg). Subjects under-reported weight at month 6 (mean difference 1.2 ± 5.5 kg) [41]. However, high positive correlations were found (Pearson’s correlation coefficient: 0.979 overall, 0.996 at Month 3 and 0.958 at Month 6).

### 3.5. Factors Associated with High BMI at Baseline

Country of residence, race, smoking variables, quality of life, weight concerns and weight confidence were all significantly associated with BMI (≥ 27 kg/m^2^) using univariate analysis at a probability threshold of < 0.10 (Table 3). When these variables were entered into the multivariate logistic regression model, six independent variables were retained. High cigarette consumption (≥ 20 cigarettes a day), having made a quit attempt in the 3 months previous to baseline, black race and weight concerns identified by a high Borrelli score were all associated with a higher risk of having a BMI ≥ 27 kg/m^2^, whereas living in France and superior quality of life were associated with a reduced risk (Table 4).

### 3.6. Factors Associated with Baseline Weight Concerns

Country of residence, gender, marital status, age, BMI, quit attempts in the 3 months previous to baseline, nicotine dependence, quality of life and weight confidence were all significantly associated with weight concerns as measured by a score of ≥ 5 on the Borrelli scale at a probability threshold of < 0.10 in the univariate regression analysis (Table 3). When these variables were entered in the multivariate model, five independent variables were retained: female gender, living in France and high BMI were associated with an elevated risk of a score of ≥ 5 on the Borrelli scale for post-cessation weight concerns, whereas older age and higher confidence to prevent post-cessation weight gain were associated with a lower risk (Table 4).

### 3.7. Factors Associated with Baseline Confidence to Prevent Weight Gain

In the univariate regression analysis, most demographic variables, BMI, cigarette consumption, nicotine dependence, quality of life and weight concerns were all significantly associated at a probability threshold of < 0.10 with confidence to prevent post-cessation weight gain (Table 3). In the multivariate model, seven independent variables were retained. Higher educational level, higher income and better quality of life were associated with a higher confidence about weight control, whereas living in the UK, female gender, severity of nicotine dependence and a high Borrelli score for post-cessation weight concerns were associated with lower confidence (Table 4).

### 3.8. Factors Associated with Weight Gain during the Follow-Up Period

The baseline variables associated with the likelihood of significant weight gain (≥5%) were assessed by univariate analysis comparing the 175 subjects who gained significant weight with those who did not (n=958). This identified demographic variables (country of residence, income, gender and marital status) and quality of life as the only baseline variables associated with significant weight gain (Table 5). In addition, a highly significant association (*p*<0.0001) was observed between weight gain and the number of cigarette-free days. In the multivariate model, being single, number of cigarette-free days, and low income were retained as associated with a higher risk of significant weight gain (Table 6).

### 3.9. Impact of Body Mass Index and Weight Concerns on Smoking Cessation Attempts

The proportion of subjects who attempted quitting smoking during the observation period was determined according to BMI and weight concerns at baseline.

## 4. Discussion

This report describes the first cross-sectional and longitudinal data obtained from the ATTEMPT cohort on weight and smoking cessation. Logistical regression analysis was used to identify factors associated with high BMI, smoking-related weight concerns, confidence about managing post-cessation weight gain at baseline in a multinational sample of 2,009 smokers intending to quit and surveyed with an Internet-based questionnaire. Factors associated with actual weight gain were evaluated in 1,28 4 followed up for six months, and the relationship between BMI, post-cessation weight concerns and quit attempts was investigated.

Concerning BMI at baseline, 1,006 subjects had a BMI of ≥ 27 kg/m^2^ (median value) at baseline. These subjects with high BMI were over-represented among heavy smokers (≥ 20 cigarettes/day), Blacks, and those subjects with a high post-cessation weight concern score, and under-represented in subjects living in France and those with better quality of life. The lower prevalence of obesity in France compared to the other participating countries and a higher prevalence in Blacks is well characterized in epidemiological studies [6,44]. One might raise the hypothesis that the rather low prevalence of blacks in France could account for this double association. However, in our study the prevalence of blacks was comparable in France, Canada, and U.K.; the lower prevalence of overweight was not found in the. two latter countries. The association between BMI and weight concern is consistent with a previous study by Pomerleau *et al*. [29] using a different single-item measure. This relationship is however debated [28].

Apart from BMI, weight concerns (score ≥ 5 on the Borrelli subscale) were more than twice as frequent among female subjects compared to males, as has been reported in several previous studies [17,28,36], whereas their frequency declines with increasing age. Living in France was related to higher weight concern scores than in other countries. This particular combination of a low BMI and high weight concerns in France is likely to be a cultural specificity. As could be expected, weight concerns were associated with low confidence in managing weight gain following smoking cessation. Low confidence was also associated with low socioeconomic status, female gender, severe nicotine dependence, low quality of life and living in the U.K.

In terms of significant weight gain over the six-month observation period, the only associated variables in the multivariate model were being single, having a low income, and longer duration of abstinence from cigarette. The latter relationship has previously extensively been described [2,13,14,16,45]. The greater weight gain in females compared to males has also been demonstrated [17,18]. However, this relationship was only found in the univariate model of our study. It is noteworthy that baseline weight concerns and confidence in preventing weight gain were not associated with weight gain in this study. Other authors have shown a positive association between weight concerns and post cessation weight gain [24]. In this clinical trial, in which smokers were probably more committed to smoking cessation, all subjects made an attempt to quit smoking. The rather low rate of smoking attempts in our study might account for the lack of association between weight concerns and weight gain.

In subjects with a high BMI, the probability of making a quit attempt was higher when weight concerns were low. The link is well documented, although not specifically in overweight smokers [3,26,28]. In contrast, the probability of making a quit attempt was higher in smokers with high weight concerns when BMI was < 27 kg/m^2^. One could assume that smokers combining high weight concerns with a low BMI have a higher general health awareness level, facilitating quit attempts [31].

Given the impact of smoking-related weight concerns on smoking cessation attempts, management of these concerns may be a useful adjunct to public health strategies aimed at encouraging smokers to stop. This is a particularly important issue in women, in whom such concerns are more important. However, studies of classical weight-control counselling programmes have not shown these to be of particular use [45–47]. On the other hand, cognitive behavioural therapy to reduce weight concerns has been demonstrated to be effective on continuous abstinence in women with weight concerns wishing to stop smoking [48].

The study has some limitations. Firstly, the inclusion criteria required subjects be willing to quit in the next three months. Such individuals may be more concerned about health problems and their weight concerns may thus not be representative of the general population. Secondly, follow-up data at six-months was only available for around half of the subjects originally included. Lost to follow-up subjects differed from the completers in terms of lower nicotine dependence level and higher BMI. Our results may have been biased by this baseline difference in lost to follow-up subjects. In-home verification showed subjects tended to under-report weight at month 6, although correlation between self-reported and verified measures was very high. It is not clear how this small distortion could have affected our results. Current data from the ATTEMPT study come from an interim analysis, and future data should allow the influence of weight concerns on successful smoking cessation attempts and long-term weight changes to be evaluated.

In conclusion, these data show that smoking-related concerns about weight gain are frequent in smokers anticipating giving up in the near future, particularly in women and in those with higher BMI, and that these concerns can influence the likelihood of making a smoking cessation attempt. Reducing concern about post-cessation weight gain in overweight smokers may be an effective strategy in encouraging more smokers to quit.

## Figures and Tables

**Figure 1.**
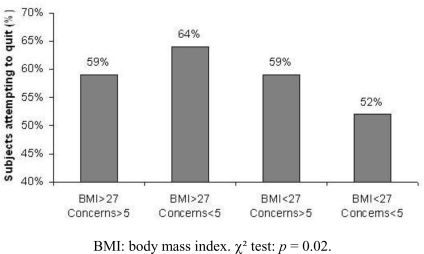
Relationship between body mass index and post-cessation weight concerns at baseline and quit attempts during the following six months.

**Table 1.** Baseline demographics.

Characteristic	Total (n=2,009)	Canada (n=208)	France (n=201)	U.K. (n=200)	U.S. (n=1,400)
Mean age (years)	47.9 (SD=8.3)	46.9 (SD=7.3)	45.5 (SD=7.4)	46.2 (SD=7.8)	48.6 (SD=8.5)
Ethnicity (% white)	90	94	97	97	89
Marital status (% married)	53	51	48	54	54
Education (% 14 or more years of formal education*)		90%*	55%	52%	99%*
Employment status (% full-time, part-time or self-employed)	67	68	66	68	66

* In the United States, formal education was reported as high school graduate, equivalent or higher and in Canada, formal education was reported as secondary school, graduate or higher.

**Table 2.** Smoking history and status, baseline weigh, BMI and weigh concern scores.

Characteristic	Total (n=2,009)	Canada (n=208)	France (n=201)	UK (n=200)	US (n=1,400)
Mean age when started smoking (years)	16.7 (SD=5.2)	16.3 (SD=5.4)	18.1 (SD=6.3)	16.3 (SD=4.8)	16.6 (SD=5.0)
Percent having made a quit attempt before	95	96	93	97	95
Median number previous quit attempts	4	4	3	3	4
Mean FTND score	5.2 (2.3)	4.8 (2.4)	4.7 (2.3)	4.9 (2.4)	5.4 (2.2)
Mean weight (kg)*	82.6 (19.0)	80.7 (19.2)	73.8 (15.7)	81.3 (17.1)	84.4 (19.2)
Mean BMI (kg/m^2^)	28.0 (5.7)	27.5 (5.8)	25.3 (4.7)	27.7 (5.8)	28.5 (5.8)
Mean weight concern scale	5.5 (2.3)	5.2 (2.5)	6.4 (2.4)	5.4 (2.3)	5.4 (2.3)
Mean confidence to prevent weight gain scale	5.6 (2.2)	5.7 (2.3)	5.2 (2.4)	4.9 (2.2)	5.7 (2.1)

BMI = Body mass index; FTND = Fagerström Test for Nicotine dependence.

* With exclusion of extreme weight change.

**Table 3.** Baseline variables associated with baseline BMI, weight concerns and weight confidence: univariate analysis.

Dependent variables	Baseline BMI ≥ 27	Baseline Weight concerns ≥5	Baseline Weight confidence ≥5
N	1986	1986	1986
Country	**<0.0001**	**0.0002**	**0.0001**
Education (low/middle/high)	0.51	0.43	**<0.0001**
Income (low/middle/high)	0.20	0.71	**0.0006**
Gender	0.41	**<0.0001**	**<0.0001**
Marital status (married/single)	0.41	**0.076**	0.56
Cigarettes per day (≥ 20)	**0.0013**	0.82	**0.011**
Quit attempts in the 3 months previous to baseline	**0.049**	**0.015**	0.96
Race (white/black/other)	**0.0016**	0.49	**0.097**
Age (continuous)	0.59	**<0.0001**	**0.007**
Weight concerns (continuous)	**<0.0001**	N/A	**<0.0001**
Weight confidence (continuous)	**<0.0001**	**<0.0001**	N/A
FTND (continuous)	0.11	**<0.0001**	**<0.0001**
Quality of Life (continuous)	**0.0002**	**0.010**	**<0.0001**
BMI (continuous)	N/A	**<0.0001**	**<0.0001**

FTND: Fagerström Test for Nicotine Dependence; BMI: body mass index. Significant associations (p < 0.10) used to identify variables for the multivariate model are identified in bold.

**Table 4.** Baseline variables associated with baseline BMI, weight concerns and weight confidence: multivariate analysis.

Effect	Odds ratio	95% CI
***Body Mass Index ≥ 27 kg/m^2^***		
Country (*versus* U.S.A.)		
Canada	0.828	0.604 – 1.136
France	**0.193**	**0.133 – 0.280**
United Kingdom	0.775	0.562 – 1.069
Cigarettes/day (≥ 20 *versus* <20)	**1.308**	**1.073 – 1.593**
Quit attempts in the 3 months previous to baseline (Yes *versus* No)	**1.247**	**1.017–1.529**
Race (Black *versus* White)	**1.683**	**1.072–2.641**
Weight concerns (Borrelli scale)	**1.273**	**1.218 – 1.329**
Quality of life (EQ-5D score)	**0.611**	**0.425 – 0.879**
***Weight concerns (≥5)***		
Country (*versus* U.S.A.)		
Canada	**0.686**	**0.482 – 0.976**
France	**2.664**	**1.787 – 3.973**
United Kingdom	0.736	0.506 – 1.069
Gender (Female *versus* Male)	**2.147**	**1.724 – 2.673**
Age	**0.982**	**0.969 – 0.995**
BMI	**1.097**	**1.073 – 1.121**
Weight confidence (Borrelli scale)	**0.625**	**0.590 – 0.663**
***Weight confidence (≥5)***		
Country (*versus* U.S.A.)		
Canada	0.824	0.564 – 1.203
France	0.978	0.660 – 1.449
United Kingdom	**0.410**	**0.283 – 0.594**
Educational level (high vs low)	**2.054**	**1.174 – 3.595**
Income (medium vs low)	**1.447**	**1.085 – 1.930**
Gender (Female *versus* Male)	**0.768**	**0.612 – 0.965**
Nicotine dependence (FTND score)	**0.915**	**0.870 – 0.961**
***Body Mass Index ≥ 27 kg/m^2^***		
Quality of life (EQ-5D score)	**1.565**	**1.038 – 2.359**
Weight concerns (Borrelli score)	**0.686**	**0.649 – 0.724**

FTND: Fagerström Test for Nicotine Dependence; BMI: body mass index. Data are presented as odd ratio estimates with 95% confidence intervals (CI) identified in multivariate regression analysis.

**Table 5.** Variables associated with weight increase ≥5% at 6 months follow-up: univariate analysis.

Dependent variable	Weight increase ≥5% in 6 months
N	1,284
Country	**0.033**
Income (low/middle/high)	**0.013**
Gender	**0.041**
Marital status (married/single)	**0.0008**
Quality of Life (continuous)	**0.074**
Cigarette-free days in 6 months (continuous)	**<0.0001**

All independent variables are baseline except the number of cigarette-free days in the 6 months follow-up period. Significant associations (*p*<0.10) used to identify variables for the multivariate model are identified in bold.

**Table 6.** Variables associated with weight increase ≥5% at 6 months follow-up: multivariate analysis.

Effect	Odds ratio	95% CI
***Weight increase (≥5%)***		
being single	1.46	1.01 – 2.10
30 days without smoking	1.35	1.31 – 1.52
Low income (low vs medium or high)	1.48	1.03 – 2.14

All independent variables are baseline. Data are presented as odd ratio estimates with 95% confidence intervals (CI) identified in multivariate regression analysis.
